# Patient decision aids: a content analysis based on a decision tree structure

**DOI:** 10.1186/s12911-019-0840-x

**Published:** 2019-07-19

**Authors:** Alexandra Gheondea-Eladi

**Affiliations:** Research Institute for Quality of Life, Romanian Academy, Calea 13 Septembrie, nr 13, Bucharest, Romania

**Keywords:** Patient decision aids, Decision tree analysis, IPDAS

## Abstract

**Introduction:**

This paper presents the preliminary results of a decision-tree analysis of Patient Decision Aids (PDA). PDAs are online or offline tools used to structure health information, elicit relevant values and emphasize the decision as a process, in ways that help patients make more informed health decisions individually or with relevant others.

**Method:**

Twenty PDAs are randomly selected from the International Patient Decision Aids Standards (IPDAS) (https://decisionaid.ohri.ca/AZlist.html) approved list. An evaluation tool is built bottom-up and top-down and results are described in terms of communicating uncertainty, completeness of the decision tree, ambiguous or misleading phrasing, overall strategies suggested within personal stories.

**Results:**

Twelve of the analyzed PDAs had branches of the decision tree which were not discussed in the tool and 6 had logically ambiguous phrasing. Many tools included dichotomous options, when the option range was wider. Several options were clustered within the “Do not take/Do not do” option and thus the PDA failed to provide all comparisons necessary to make a decision. Some tools employ expressions that do not differentiate between lack of information and known negative effects. Other tools provide unequal amounts or non-comparable bits of information about the options.

**Conclusion:**

These results indicate a very loose range of interpretations of what constitutes an option, a treatment, and a treatment option. It thus emphasizes a gap between theory and practice in the evaluation of PDAs. Future developments of PDA evaluation tools should keep track of missing decision tree branches, accurate communication of uncertainty, ambiguity, and lack of knowledge and consider using measures for evaluating the completeness of the option spectrum at an agreed period in time.

**Electronic supplementary material:**

The online version of this article (10.1186/s12911-019-0840-x) contains supplementary material, which is available to authorized users.

## Background

Patient Decision Aids (PDA) are decision support systems that help patients decide by providing information that is purposefully structured for decision-making. Behind any decision support system there is an abstract representation of the decision-making process, called a decision-tree. In this paper we argue that most PDAs have missing decision-tree branches, many have logically ambiguous statements or include only 2 of many options, thus re-framing the decision by providing an incomplete context.

### What are PDAs?

PDAs are online or offline tools used to structure health information and elicit relevant values in ways that help patients make more informed health decisions individually or with relevant others [1, 2]. Their design is based on the idea that informed decisions improve when information is structured and the decision process is emphasized [3–5]. PDAs differ from Patient Information Resources (PIR) in that they not only inform patients, but also help them elicit their values and preferences and may include information about the decision-making process, such as a need to search for more information or to delay the decision [6]. In doing so these tools may be tailored to accept user’s input (called *interactive* PDAs, such as the one available here: [7]) or the values and constraints of relevant others (family or two person PDAs, such as the one available for deciding on a place of care [8]). A list of approved PDAs can be found at [9].

PDAs are underpinned by Shared Decision-Making (SDM), a philosophy of doctor-patient communication and the new emerging gold-standard in healthcare [10]. SDM is usually a three-fold intervention aimed at activating patients to ask questions, training health and medical professionals to inform and include patients’ values in treatment decisions [11–18] and using patient decision-support systems before, during and after doctor-patient consultations [16, 18–23]. SDM is underpinned by the ethical philosophy of patient autonomy and right to make an informed decision [20, 24–28].

### Why have PDAs been used?

PDAs have been designed to improve and standardize the quality of doctor-patient communication in order to allow patients to make more informed health-related decisions [1, 29–31]. Three main problems of doctor-patient communication are meant to be addressed in any PDA: the knowledge gap between doctors and patients in order to facilitate patient activation (asking questions, understanding doctor’s answers, etc.) [32–35], individual differences in information delivery during consultations [20, 36], and the lack of information about the decision-making process behind the health condition or screening [6, 37]. Thus, PDAs are aimed at providing the following solutions: they locally bridge the knowledge gap between doctors and patients enough to make a common-sense discussion possible; they standardize the transmission of information in doctor-patient interaction; they offer a longitudinal process-based perspective, rather than a static resolution-based perspective on health-related patient decisions. PDAs are not artificial intelligence tools designed to make a decision for the patient. They structure the information, elicit patients’ values and reveal the steps of the decision process in a way which makes an informed decision possible.

### When and how are PDAs used?

PDAs are used for health concerns which have multiple possible approaches, options or treatments, and are ethically required to include the option not to take any treatment (see 27–29 for a bioethical discussion about this). When a health concern has a single known solution, it is nevertheless possible to build a PDA which helps the patient decide whether to choose the option available or not and to understand the consequences of this decision. PDAs may be used before, during or after doctor’s consultations, depending on their purpose and design.

### Why improve PDAs?

The study of PDAs is very important because they have the potential to bridge various gaps between doctors and patients. First, doctor’s consultations take very little time [38], which translates either into relatively little time for addressing patients’ questions or into leaving out information such as self-management and complementary or alternative medicines (CAM) [39, 40]. Second, patients increasingly try to bridge the doctor-patient knowledge gap by searching for health information online [41–44]. With low research skills [45–47] and low health literacy in approximately 50% of the US population [48] and 47% of the European population [49], searching for health information online can lead to mis-information. Therefore, developers and medical practitioners, healthcare providers and decision-making specialists evaluate PDAs based on the extent to which they provide appropriate information and the relevant structure for decision-making.

This paper, therefore, aims to contribute to the improvement of PDA content development and structure. In the following sections we review the main decision-making theories that inform content structure for PDAs, and we present the results of a content decision-tree analysis of 20 randomly chosen PDAs. A custom-made decision-tree evaluation tool is described which includes both bottom-up and top-down indicators. Results will reveal how many of the PDAs have missing decision-tree branches, hinder option comparison and have logically ambiguous phrasing. The results are then discussed with respect to possible explanations, solutions and future research.

In the next subsections we show that completeness and option comparability of the PDA decision-tree, as well as the logical ambiguity of PDA contents are not thoroughly addressed through the currently available PDA evaluation criteria. With this aim, we first review current PDA evaluation criteria and tools. Then, we show why analysing the content structure of PDAs from the proposed points of view is important.

### Current PDA evaluation criteria and tools

PDAs are evaluated based on their development process and their impact. At development level, the International Patient Decision Aids Standards Collaboration (IPDAS) includes evaluation of the following domains: “systematic development process; providing information about options; presenting probabilities; clarifying and expressing values; using patient stories; guiding/coaching; disclosing conflicts of interest; providing internet access; balanced presentation of options; using plain language; basing information on up to date evidence; and establishing effectiveness” [50], p. 1. These variables are measured a priori.

The impact of PDAs has been assessed with respect to different constructs like health outcomes, quality of the decision-making process, quality of care and value-congruence of decision. These are measured a posteriori.

*Health outcomes* include “values clarity, decision certainty, decision regret, confidence, desire for participation in decision, question asking, actual participation in decision, communication quality (information provision/receipt, good processes of communication/care, satisfaction with communication/decision/care” [51], p. 3.

*The quality of the decision-making process* [52] is evaluated based on the extent to which patients have a clear formulation of the decision problem (measured by the Preparation for Decision Making Scale [32, 53], feel informed about the options, risks, benefits and consequences (measured by a subscale of the Decisional Conflict Scale [54]), feel they know their values, are actively involved in their care decision (measured by the Perceived Involvement in Care Scale [55] based on their preferences (measured by the Control Preferences Scale [14]).

Q*uality of choice* is operationalized as “the extent to which patients are informed and receive treatments that reflect their goals and treatment preferences” [52]. This is an objective assessment of patient’s knowledge of the options and outcomes and the concordance of the chosen option and what matters most to the patient [52]. The last aspect of quality of choice is also called v*alue congruence of the decision* [1, 2].

Although the IPDAS requirements are minimal [50], their ambiguity has provided grounds for criticism [51]. On the other hand, process-based evaluation criteria have been criticized for overlooking improvements in patients’ quality of life [56]. In this paper we argue that, for the moment, PDAs also have problems accurately representing and presenting the decision tree underpinning their content structure. Moreover, we argue that the PDAs analysed represent incomplete (or clustered), partially comparable decision trees which also have logically ambiguous phrasings.

### Why consider completeness, comparability and logical non-ambiguity to evaluate PDA content?

In general, content structure in PDAs is important for PDA developers because it directly influences the resolution of the decision-making process. Incompleteness, non-comparability and logically ambiguous phrasing are content structure characteristics which lead to biased decisions, by definition [57–59]. Incompleteness of the decision tree means that there are options which have not been presented or are somehow concealed [57]. Non-comparability means the criteria used for evaluating options are not the same for each option [57, 59]. Logical ambiguity means that the phrasing does not allow the user to make a clear inference based on the information provided. While incompleteness and non-comparability are characteristics of the decision-tree underlying the content structure [57], logical non-ambiguity is a characteristic of the content itself. However straightforward this may seem, evaluation of contents based on these criteria is not trivial.

There are three main theories which explain the influence of content structure on the resolution: descriptive, normative and predictive theories of decision. The descriptive theories of decision may support a certain content structure based on how patients decide and what their decision-making needs are [6, 37, 60, 61]. Normative decision theories may support a certain content structure to avoid the risks associated with cognitive traps [61, 62] or with ethical concerns [51, 63, 64]. Predictive theories of decision relate current content structure to patients’ future decisions. These may argue that a certain content structure will make patients choose one option more often than others, irrespective of what would actually meet patients’ needs [65, 66]. Decision support systems are expected to attenuate the shortcomings emphasized by descriptive theories of decision-making behaviour and employ evidence-based normative theories in order to avoid predicted hazardous decisions.

Decision tree analysis is based on normative theories of decision and has long been used in medical decision-making [67–70], as well as in other disciplines [71]. For PDA development and evaluation, a decision tree is an abstract structure which transforms information into knowledge by providing the following structure [57]:

- What is the decision?
- What are the options (including the option to do nothing)?
- What are the expected outcomes of each option?
- What are the probabilities of each expected outcome?
- Which options have unknown outcomes and why are they unknown (research has not been performed; research is under way; research results are contradictory, no research has been undertaken so far, etc.)?

Based on a rational choice theory, there are also second-order characteristics that the decision tree should have [57, 72]. Two of these are particularly important for the analysis in this paper:

- The completeness of the option range;
- The comparability of information across options.

The use of a decision tree analysis assumes that the decision-maker is autonomous [12, 63, 73, 74]. From this point of view, PDAs should address the needs of patients, surrogate decision-makers, as well as family members or relevant others considered important by the decision-maker.

Normatively, PDAs should avoid the following traps:

- partitioning the options, since decisions are context dependent; for example, by listing only a part of the available options, instead of all the available options, certain options may seem more appealing than in the complete context.
- presenting detailed outcomes of only one part of the options; for example, by providing expected frequencies for treatment outcomes, but not for the option of not taking any treatment.
- presenting detailed outcomes for taking the treatment for short periods of time, when patients are usually expected to take the treatment for much longer periods of time. For example, presenting success rates and side-effects for taking contraceptives for one year, when most women are expected to take them for 5 to 10 years.
- presenting unknown information as lack of effect; for example, by reporting that “studies have not shown the effects” or that “there are no studies showing that...”, when studies have not been performed on that particular treatment.

From a descriptive point of view, there are further criteria which are relevant for the quality of the decision. For example, PDA developers should avoid suggesting overall strategies that decision-makers use to avoid deliberation. These are usually described by the literature on heuristics and biases [62, 75, 76], small sample decisions [77, 78], emotional decisions [79–81] and custom based or social norms based decisions [82–84]. Such strategies fundamentally change the decision-tree and simplify it to suit cognitive limitations.

Based on these theories, it is possible to propose the following definition: an informed decision is a decision in which all branches of the logical decision tree are openly discussed. Moreover, if all branches of the logical decision tree are described in a complete and comparable manner together with the patient’s values and the steps of the decision-making process, then the decision is considered informed. When some branches have unknown information, the decision is considered informed if the lack of information is communicated clearly. If some branches are not known or are clustered in ways that do not allow full comparisons, or if it is unclear where there is incomplete knowledge, the decision is not informed. Also, the decision is considered informed, if the steps in the decision-making process needed in order to acquire more information are fully and openly discussed.

To sum up, based on both a decision theory driven definition of the informed decision and the ethical principle of patient autonomy, it is possible to suggest that the following criteria are used for PDA evaluation:

1. Informed decision:
  - Decision tree:
    i. Openly signalling incomplete knowledge
    ii. Identifying missing decision tree branches
    iii. Controlling that criteria in the Pros and Cons section is comparable
    iv. Controlling that criteria in the Pros and Cons section are expressed in logically non-ambiguous statements
  - Content
    i. Expressing information content in logically non-ambiguous statements
  - Decision process
    i. Controlling overall strategy reports in Personal Stories
    ii. Presenting the set of Personal Stories in ways which do not balance the choice toward a certain option
2. Autonomy
  d. Decision process
    i. Developing PDAs in ways which allow a Shared Decision to be made with relevant others, including the doctor
    ii. Developing PDAs in ways which allow a Shared Decision to be made if the intended users are Surrogate decision-makers

## Methods

In this section, an evaluation tool is built bottom-up (based on grounded theory methodology [85, 86] which elicits variables driven by the particularities of the PDA content analysed) and top-down (based on relevant theories of decision-making identified in the Background section). We present the sample and operationalization of bottom-up and top-down indicators such as communicating uncertainty, completeness of the decision tree, ambiguous or misleading phrasing, and overall strategies suggested within personal stories.

### Sample

The IPDAS currently holds a list of 34 PDA developers that provide free access to their IPDAS compliant tools [87]. A total of 337 PDAs have been developed in this way [1]) at the time the analysis was made.

Twenty online tools have been randomly selected from the IPDAS accepted PDA list in order to construct the evaluation criteria (see the sample in Table 1). Only one tool from the sample could not be evaluated because it was not free for use and required international ordering.

**Table 1:** The PDA random sample

ID	Subject	Title	Valid	Current as of	Author	Recommended use
1.	Alzheimer’s Disease	Alzheimer’s: Consider options for long-term care. Mayo Clinic [88]	Yes	25/03/16	Mayo Clinic	Ø Patients and Consumers
2.	Back Pain	Low Back Pain: Should I Try Epidural Steroid Shots? Healthwise [89]	Yes	23/05/16	Healthwise	!Patients and Consumers
3.	Breast and Ovarian Cancer	Breast Cancer Risk: Should I Have a BRCA Gene Test? Healthwise [90]	Yes	26/07/16	Healthwise	!Patients and Consumers
4.	Breast Cancer^a^	Reducing the Risk of Breast Cancer With Medicine: A Guide for Women Agency for Healthcare Research and Quality (AHRQ) [91]	Yes	01/01/10	Agency for Healthcare Research and Quality	# Patients and Consumers
5.	Breast Cancer	Understanding ductal carcinoma in situ (DCIS) and deciding about treatment. University of Sydney [92]	Yes	2010	National Breast and Ovarian Cancer Centre	Ø During consultation
6.	Cholesterol	Cardiovascular Risk Decision Support Tool. HealthDecision [7]	Yes	2015	Jon Keevil, MD, HealthDecision	!During consultation
7.	Connective Tissue Disorders	Dupuytren’s Disease: Should I Have Hand Surgery? Healthwise [93]	Yes	24.10.2016	Healthwise	!Patients and Consumers
8.	Elbow Injuries and Disorders	Tennis elbow: Should I have surgery? Healthwise [94]	Yes	23.05.2016	Healthwise	!Patients and Consumers
9.	Flu (Influenza)	Flu: Should I take antiviral medicine? Healthwise [95]	Yes	23.05.2016	Healthwise	!Patients and Consumers
10.	Osteoarthritis	Knee osteoarthritis: treatment options Option Grid Collaborative [96]	Yes	2014	OptionGrid	!Patients and Consumers
11.	Osteoporosis	Should I take risedronate (Actonel®) for osteoporosis? Cochrane Musculoskeletal Group [97]	Yes	2011	Cochrane Musculoskeletal Group	Ø Doctor Consultation
12.	Panic Disorder	Panic disorder: Should I take medicine? Healthwise [98]	Yes	26.07.2016	Healthwise	!Patients and Consumers
13.	Prediabetes	Prediabetes: Which Treatment Should I Use to Prevent Type 2 Diabetes? Healthwise [99]	Yes	23.05.2016	Healthwise	!Patients and Consumers
14.	Prostate Cancer	Prostate cancer: Should I choose active surveillance? Healthwise [100]	Yes	26/07/2016	Healthwise USA	!Patients and Consumers
15.	Prostate Cancer	Prostate specific antigen (PSA) test: yes or no? Option Grid Collaborative [101]	Yes	19/09/2016	OptionGrid Collaborative	!Patients and Consumers
16.	Prostate Diseases	Enlarged Prostate: Should I Take Medicine? Healthwise [102]	Yes	14/06/2016	Healthwise	!Patients and Consumers
17.	Sore throat	Sore throat: antibiotics or not? Option Grid Collaborative [103]	Under revision	14/07/2014	Option Grid Collaborative	!Patients and Consumers
18.	Toilet Training	Bed-wetting: Should I do something about my child’s bed-wetting? Healthwise [104]	Yes	26/07/2016	Healthwise	!Patients and Consumers
19.	Testicular cancer	Testicular Cancer: Which Treatment Should I Have for Stage I Nonseminoma Testicular Cancer After My Surgery? Healthwise [105]	Yes	26/07/2016	Healthwise	!Patients and Consumers
20.	Care	When you need extra care, should you receive it at home or in a facility? [8]	Yes	2010	Ottawa Hospital	Ø Patients and Consumers

^a^Not a free tool

Legend:! = interactive tool; Ø = not interactive; # = unavailable tool

From the sample, there were 17 interactive tools (i.e. they accept input information from patients, such as blood test results or other relevant information) and 2 which included sections on or openly discussed sharing the decision with other parties beside the doctor (support groups, psychologists, family, etc.). Also, 3 tools were only available in PDF format, 13 as Web Pages and 4 in several formats (Web and PDF).

The tools analysed can be divided into four types, based on the way they structure the decision-making process (see Decision aid type in Additional file 1):

- eligibility, risk assessment, decision, documents [9]
- knowledge, pros and cons [9]
- knowledge, values, resolution [13]
- question structure [9]
- test, information, re-test, decision [2]

The structure of the decision-making process depends on the organization that developed the tool.

### Data analysis and operationalization

Data analysis was performed in two steps. First, a core set of top-down criteria has been created to assess the decision-tree first-order components and second order characteristics. Secondly, grounded evaluation criteria were added to the evaluation grid based on the characteristics of each tool. At the end of this analysis, all tools would be evaluated against all the criteria (see Additional file 1 for the data analysis database). No additional criteria have emerged after the 11th tool analysed and a total of 37 criteria have been built in this way. The criteria constructed in this way are provided in Table 2 and Table 3. Based on the review presented in the Backgound section, they are clustered into three main categories: decision tree, decision process and content.

**Table 2:** Top-down operationalization

Criteria	Definition
Decision tree	
Incomplete knowledge acknowledged	Does the PDA include acknowledgement of decision nodes with incomplete information? Does it emphasize the areas where knowledge is incomplete
Missing structure 1 and 2	Does the tool investigate all logical branches of the decision tree? If not, describe missing structure.
Pros and Cons logic	Does the “pros and cons” list miss any logical branches of the decision tree? If yes, describe missing structure.
Decision process	
Overall strategy in Personal Stories (P.S)	Do the stories reflect an overall strategy for choosing treatments? E.g. “Try out anything”, “Choose only treatments with less side-effects”, etc.?
Biased stories	Does the Personal Stories Section include more reports of one treatment option than the other
Decision-aid type	Description of the structure of the decision-making process in terms of steps taken to reach a decision
Shared Decision	PDA contains elements that allow taking into consideration the opinions of other parties (family members, significant others)
Surrogate awareness	In case the text is used by surrogate decision makers, does the PDA include reference or suggestion to consider the values of dependent patients (older patients, low literacy patients, children, etc.) and not just those of the surrogate
Content	
Logically ambiguous phrasing	Does the PDA include sentences or statements which may be misleading or ambiguously phrased. If yes, include phrase.

**Table 3:** Bottom-up operationalization

Criteria	Definition
Decision tree	
Probable/Certain	Represents option outcomes in probable or certain terms
Frequencies	Tool uses frequencies
Percentages	Tool uses percentages
Includes significance estimate	Background information presented includes insignificance estimates
Number of Options	Number of options presented by the tool
Clustered Options	Are multiple options clustered into a single one?
Decision process	
Interactive	Tool accepts user input
Related PDA	Are there other tools which evaluate further possible treatments to the same problem, which could be used together to create a more balanced representation of the available options
Section on what experts recommend	Tool includes a section on what the experts recommend
Cause investigation	Tool suggests investigation of the cause of symptoms
Content	
Personal stories (PS)	Tool includes personal stories
No. of PS.	Number of personal stories
Changing order of PS.	Order of stories changes each time tool is accessed
Story 1–4 Reported outcomes	Reported outcomes in story 1–4
Story 1–4 Reported Side-effects	Reported side-effects in story 1–4
Story Reported Causes	Do the stories reflect possible constraints/causes that have led to/facilitated the health situation experienced
Includes quality assessment of the scientific evidence (GRADE)	Background information presented includes quality assessment measures of the scientific evidence (e.g. GRADE levels)

Decision tree criteria refer mostly to the elements of the tree and their second order characteristics, like option range completeness, comparability and incomplete or complete knowledge.

The Decision tree category includes three criteria: Incomplete knowledge acknowledged, Missing structure, and Pros and Cons logic.

Incomplete knowledge acknowledged is a criterion which indicates whether there is insufficient information about a tree node. It proposes that PDAs should emphasize cases in which information currently available in the decision tree is incomplete, that is either uncertain (expressed usually in probabilities of success, false-positive, false-negative probabilities, etc.), ambiguous (e.g. if work is currently being undertaken to find a suitable treatment option or if research results are too contradictory or non-comparable to each other to release probabilities of success, etc.) or unknown (e.g. if no study has ever been conducted). Good performance in this case would be to have PDAs which indicate not just the quality of the literature reviewed in the development process, but also clearly state cases in which nothing is known about this option (e.g. there has been no research undertaken on this topic). In this case, the lack of research should be clearly delimited from negative result cases (e.g. research has shown that …, but did not show this …) and from contradictory results (e.g. some research has shown this and some research has shown that, based on the same or comparable research design).

Missing structure is a criterion aimed at checking if there are any missing branches in the PDA decision tree, by comparing the decision tree which emerges from the PDA with the logical decision tree derived from it. The PDA decision tree can be revealed for any PDA by laying down the options, sub-options, the probabilities and so on. This abstract structure is laid out only for the options and the information provided in the PDA content and possibly the first order logically accurate inferences based on it. Based on this structure, the complete and comparable logical decision tree can be constructed. This is the standard of comparison for the PDA decision tree based on which the criterion Missing structure is evaluated.

Pros and cons logic is a criterion which is also based on the construction of the decision tree, but only the one which emerges from the pros and cons section in the PDA. As in the case of the Missing structure criterion, based on the pros and cons decision tree, a complete and comparable logical decision tree is constructed as the standard for comparison. Differences or imbalances in the information provided for the pros and cons decision tree and the logical one are described and counted in the Pros and cons logic criterion.

The Decision process section includes four criteria: Overall strategy in Personal Stories, Decision-aid type, Shared Decision, Surrogate awareness.

Overall strategy in Personal Stories describes the cases in which personal stories suggest that the person has employed a certain strategy over the entire decision tree. Strategies like “Try out everything” or “Choose only treatments with less side-effects” or “I had heard about this” implying the use of the accessibility bias and so on are likely to help people choose a strategy, and not necessarily to make a choice. Some strategies help elude the deliberation and value elicitation process, but the positive or negative nature of this situation cannot be judged irrespective of the resolution content. In some cases it may completely bypass information which could be relevant, in others it may be an indicator of how others have decided. There is no example of good practice in this case, since the influence of overall strategies depends mostly on their health-related consequences. This is why, in this analysis we are only interested in controlling this variable.

Biased Personal Stories refer to the use of another type of heuristic, which can be derived from the personal story section, and that is the frequency with which others have chosen a certain treatment. If the personal story section includes a total of four stories and three of them discuss the same treatment option, a decision-maker may deduce that this is the most frequently chosen option and be influenced by it or, on the contrary, deduce that this is what the designer or funding body of the PDA would prefer and choose the one less favoured. Good practice in the use of personal stories should include equal frequencies of each option [106].

Shared Decision and Surrogate Awareness are two criteria which verify whether the values and choices of other relevant people are taken into consideration, either directly (Shared Decision) by providing separate space for their preferences, options, values or choices, or indirectly, by suggesting that their preferences, etc. be taken into consideration. In this case, good practice varies from problem to problem (Surrogate Awareness).

The Content category includes a single criterion: Logically ambiguous phrasing. This criterion describes and counts the cases in which a phrase or sequence of sentences does not allow a clear-cut judgment to be made or opens up several logical possibilities which hinder the accuracy of the inference made based on this phrasing.

The bottom-up criteria can also be clustered into the same categories as before, pointing to the decision tree, process and content (Table 3). From the Decision tree category, the criterion which requires further explanation is **Clustered Options.** This criterion counts the cases in which several options are presented as a single option, as visible in the PDA section called “What are my options?” or in the Pros and Cons Section. As discussed earlier, such clustering leads to the impossibility of consistently comparing all options and it may thus bias the decision [69].

All indicators have dichotomous answers (yes/no), except the following: “Probable/certain”, “Story 1-4 Reported Outcomes”,” Story 1–4 Reported Side-effects”, “Overall Strategy in Personal Stories”, “Story 1-4 Reported Constraints”, “Decision-aid type”, “Phrasing problems“, “Missing structure 1 and 2”.

## Results

The data analysis revealed the following:

- 12/20 PDAs have missing decision tree branches;
- 8/20 PDAs cluster several options into a single one;
- 12/20 PDAs have logically ambiguous phrasing;
- 4/20 PDAs have missing or unclear information in the pros and cons section;
- 1/20 presented frequency measures with significance estimates;
- 1/20 PDA has a clearly biased personal story section.

In the following section the main results will be presented with respect to each relevant indicator. The numbers presented between block parentheses, e.g. [n], represents the number of the PDA given in Table 1. All results presented are based on the database in the Additional file 1.

### Missing decision-tree branches

#### Including dichotomous options, when the option range is wider

For example, in tool [5], the options are presented as dichotomous, but after studying the information documents (presented only when the person rejects the medication) it is clear that there are more options clustered in the Do-not-take-medication option.

#### Including only certain options, but omitting others

For example, in tool [13] there is no option for taking only metformin (medication). The results presented include: 1) major lifestyle changes; 2) metformin and lifestyle changes; 3) placebo and lifestyle changes. It is unclear if there is no evidence for taking only medication with no lifestyle changes (although this may be a logical and possibly convenient option) or if the studies reveal contradictory results. In tool [14] the option called “watchful waiting” is also not described, despite being mentioned as different from “active surveillance”. Also, in tool [20] the option of assisted living (Gawande 2015) is not included, despite the fact that this service is also available in the country in which the tool was designed for.

#### Missing options in the final decision

In tool [2, 6] and in others developed by Healthwise there is no “do nothing” option for the question: “Check what you need to do before you make this decision”.

#### No presentation of side-effects replaced with suggestion to discuss with the doctor

One of the analysed tools did not discuss the side effects of radiotherapy, mastectomy or lumpectomy [4]. In the case of radiology it states that side effects should be discussed with the doctor. Side effects are a very important attribute of patient decision-making and very much relevant for this treatment decision.

#### Providing information about false-negative or false-positive results, but not both

For example, in PDA [2], a discussion about false-negative results was included, but there was no discussion about false-positive results.

Overall, missing structures have been identified in various ways, as presented in Table 4. Qualitative reports on each case are available in the Additional file 1.

**Table 4:** Frequency distribution of variables used to identify missing decision tree branches

	Yes	No	NA	No available data
Related PDA	5	13	1	1
	Missing	Clear	NA	No available data
Pros and Cons logic	5	10	4	1
	Yes	Possibly yes^a^	No	No information available
Missing structure 1	10	1	7	1
Missing structure 2^b^	6	1	8	1
Missing structure 1 OR Missing structure 2^c^	12	2	–	–
Missing structure 1 AND Missing structure 2^c^	–	–	7	1

*NA* Not applicable

^a^Indicates lack of discussion about cause which could potentially change the options, either add more or have less

^b^Does not add to 20 because each PDA was evaluated by 1 or 2 missing structure variables

^c^Does not add to 20 because values are based on contingency tables

### Comparability of options

#### Failing to consider treatment time

In the tool for Dupuytren’s Disease [6] there is no discussion on the risks of collagen injection on the long term. If lack of knowledge on this aspect is the reason for this, then this should be emphasized.

#### Omitting decisions based on the cause of symptoms

Perhaps the most intuitive example is given by tool [17], where the treatment depends on the cause of the “sore throat” symptom. Consequently, a useful decision-making structure would provide advice on how to see what caused the sore throat and then recommend treatment there off. There is no mention that it is not possible to know what caused the sore throat or that it is too expensive, or any line of thinking which generated the options. Only 2 PDAs discuss the cause of symptoms, while 15 of the 20 PDAs analysed do not (Table 5).

**Table 5:** Frequency of PDAs framed in terms of the cause of symptoms

	Yes	No	NA	No data available
Cause of Symptoms	2	12	5	1

#### Not giving the same details about the option “Do not take any treatment”

For example, the line “Why should you avoid type 2 diabetes” presents information in a way that is not comparable to the treatment options. Information about how many people get eye problems, nerve and kidney problems and after how long do these or does death occur would provide a more balanced approach to this ethical possibility.

The comparability problems are visible both from the missing structure variables summarised above and detailed in the Additional file 1, as well as by looking at the clustered options variable. In general, 8 out of the 20 PDAs analysed have either clearly clustered options or a possibly clustered option (Table 6).

**Table 6:** Frequency of clustered options

	Yes	No	NA	Yes/Unclear^a^
Clustered options	7	10	2	1

^a^It was unclear whether the options were clustered or not, but there is a possibility that they were. Namely, the option “lifestyle changes” includes several options like diet, exercise, sleeping patterns, etc.; do these have any effect on the health problem separately or only when taken together?

### Logically ambiguous phrasing

#### Mis-representing values, preferences and feelings

For instance, tools designed by Healthwise include a decision stage called “Your feelings” which usually is designed to elicit preferences. However, the questions in this stage seem not to differentiate between preferences, values and feelings. For example, tool [19] has a value elicitation section in which the questions are not only difficult to answer, but also have inappropriate scale measurements.

- "I want chemo or lymph node surgery for the best chance of cure at the start (Not important … Somewhat important … very important).
- "I might not need more treatment, so I want surveillance (same scale as previous);
- "I’d rather have side effects from treatment than have surveillance (same scale);
- "I can make sure I go to checkups and tests during surveillance (same scale);
- “I don’t mind banking my sperm to have treatment” (same scale).

##### Phrasing the questions

For example, what should a patient answer to the first question if lymph node surgery is an option, but chemo is not? What should a patient answer to the second question, if surveillance is desired despite the fact that treatment might be needed? Another possibility is to rephrase the questions to elicit preferences of each option discussed.

##### Scale measures

A better scale would have been “Strongly Agree... Strongly Disagree”.

##### Suggested solutions

In general, value elicitation requires a different approach. For example discussing the degree to which each option leads to loss of personal freedom or dependence on others or the degree to which each option leads to increased personal care and less care for significant others (dependent or not) and so on. If taking a pill or doing exercises for the rest of your life is seen as “being dependent”, while a short term intervention is seen as “giving freedom” or the other way around, this may interfere with the final resolution. The current form does not allow value and preference comparisons.

#### Inaccurate reporting of probability

For example, in tool [11] the frequencies are explained in an inaccurate manner: “Blocks of 100 faces show a ‘best estimate’ of what happens to 100 women …”. However, blocks of 100 faces shows what may happen to 100 women, since not every 100 women will have the same results, but 100 randomly chosen women. Another example is the phrase “There is no way of knowing in advance if you will be one of those affected” is biased because it conceals that there is no way of knowing that you will be one of the not affected. The correct version would be, for example: “There is no way of knowing in advance if you will be one of those affected or not (who break a hip or not)”.

#### Incomplete presentation of study results

Presenting what research does not show is just as biased as showing only what it does show. In tool [16] the following phrase seems difficult to use: “Some men try dietary supplements for BPH, such as saw palmetto or beta-sitosterol. But scientific studies don’t show that saw palmetto helps with urinary problems or that beta-sitosterol is safe or helps over the long term”. This brings on the question of: what do these studies show? Do they show that these supplements are not better than placebo? Do some studies show significant effects, while other studies show insignificant effects? The reader cannot draw any practical conclusions based on this presentation of results.

In general, incomplete knowledge acknowledgment has been observed in 2 out of 20 PDAs, while most of the other PDAs analysed do not acknowledge incomplete knowledge relevant to the decision (Table 7). Further medical literature reviews on all areas of the PDAs studied is required in order to establish whether this situation is due to the fact that there is incomplete knowledge over the topic or to the fact that this was not a requirement of PDA standards. However, 9 out of the 16 PDAs which did not include any incomplete knowledge acknowledgment also have missing decision tree branches (Missing structure 1 OR Missing structure 2 * Incomplete knowledge acknowledged). The rest of 7 PDAs do not have missing branches. Also, from these 16, 5 have missing Pros and Cons logic and for the rest of 11, the Pros and Cons logic is not applicable (Pros and Cons logic * Incomplete knowledge acknowledged). Only 3 of these 16 PDAs without incomplete knowledge acknowledgment have related PDAs on the same topic and 8 do not have them (Related PDAs * Incomplete knowledge acknowledged).

**Table 7:** Frequency of incomplete knowledge acknowledgment

	Yes	No	Sometimes	NA
Incomplete knowledge acknowledged	2	16	1	1

Overall, logically ambiguous phrasing has been visible in 12 out of 20 PDAs analysed (Table 8).

**Table 8:** Frequency distribution for logically ambiguous phrasing problems

	Yes	No	Na	No data available
Phrasing Problems	12	6	1	1

### Other variables

In the Personal Stories Section, 4 PDAs had reports of overall strategies suggested within the personal stories. Only 1 was clearly biased toward one of the treatment options in the sense that the majority of personal stories [2] reported the choice of one of the options (see Table 9).

**Table 9:** Frequency of Reported Strategies and Biases in the Personal Stories section

	Yes	No	Na	No data available
Reported strategy in PS	4	1	14	1
	Yes	No	Na	No data available
Biased PS	1	11	6	2

Most of the PDAs analysed did not discuss the possibility of having a shared decision, and only 2 of them were designed to allow for such a decision. From the 5 PDAs which advised the user to have a shared decision with the patient, 2 suggested it is shared with the doctor and 1 suggested it is shared with the doctor and others and 2 with the patient (Table 10).

**Table 10:** Frequency of tools adapted for shared decision making or which advise the decision-maker to share the decision with relevant others

	Yes	No	Na	No available data	
Shared Decision	2	17	–	1	
	With the patient	With doctor	With doctor and others	No	No available data
Advises the decision-maker to share the decision	2	2	1	12	1

## Conclusions

Based on a random selection of IPDAS approved PDAs, we showed that many current tools have missing branches of the decision tree, discuss only two of many options or partition the option range such that comparability is impaired and had logically ambiguous phrasing.

The main limitation of this study is the small sample on which the conclusions are based. However, the random character of the sampling method adds strength to the conclusions. Either way, the analysis can be replicated and all variables use objective measures.

Based on this analysis, there are important questions to address for future research:

1. Do dichotomous options lead to informed decisions?

Many of the PDAs analysed present only two options. Some of them analyse three or four options, but this is rare. The most troubling cases are those in which only one option is basically presented, while all the other possible options are clustered in the “Do not” option. If other options are envisaged, they are inserted in a different tool, and this does not ensure comparability of all cases.

2. Do we need to re-think the value elicitation sections?

The decision-making literature makes a very clear distinction between values, preferences and feelings. While preferences express the order of the options, values are over-arching principles which can be applied differently to specific situations (such as moral values [107]). For example, freedom is a value opposed to dependency. It can be applied in many forms, from self-management, to patient participation in treatment decisions, to choosing treatments that avoid dependency on others and so on. In other circumstances, it is not possible for a patient to understand how a general value can be applied to a particular medical situation. In such cases, a decision tool should elicit the core value, help the patient realize whether further information is needed and direct the patient towards a discussion with the medical practitioner.

3. Does emphasizing what we don’t know lead to informed decisions?

Information about what information is missing is never included in the PDAs analysed. Missing information includes discussions about the cause of symptoms in the cases in which the PDA addresses treatment for symptoms (sore throat, low back pain, etc.), but also about lack of research or inconclusive results.

### Good practice

Two examples of good practice emerge from the analysed sample. Despite the fact that these tools have their own problems, they provide some interesting solutions for two of the problems identified earlier.

- Tool [5] is a highly interactive PDA that provides risk assessments based on the patients’ blood test levels. Much information is available in pdf format. Several medicines are described in separate files, nutrition and exercise information is available. This is the only tool which presents so many options. However, because they are not presented on the same structure, comparing the treatments and their expected outcomes may prove difficult.
- Tool [13] is an example of good practice in reporting uncertainty: “Another thing to understand is that the evidence can’t predict what’s going to happen in your case. When evidence tells us that 2 out of 100 people who have a certain test or treatment may have a certain result and that 98 out of 100 may not, there’s no way to know if you will be one of the 2 or one of the 98.”

### Impact of the findings in practice

In light of these findings, there are grounds to consider a logical decision tree analysis for developing all PDAs. This recommendation is valid also for groups who perform literature reviews for PDA content development. It seems crucial that the decision tree emerging from the PDA is constantly verified against the logical decision tree, in order to identify the missing branches, clustered options, ensure completeness (at the time of the design) and comparability of options or elicit nodes where current level of knowledge is incomplete. More than this, the relationship between PDA content development and the decision tree should be one of mutual construction.

There is however a danger that even if the completeness of the decision tree is checked, some branches will remain concealed in two ways. First, an internally consistent (a perfectly comparable and complete decision tree with revealed areas of incomplete knowledge) but externally inconsistent PDA may create the illusion that the search for new options is not needed. Without a critical eye these criteria will not guard against internally consistent, but externally incomplete PDAs. Second, these criteria are not helpful if the end-goal of the decision is not also openly revealed. For example, to avoid this danger, PDAs could openly state whether the end goal of the PDA was to increase quality of life, cure the illness and/or to make a value-driven decision, all of them or something else.

## Discussion

In view of the results presented, it is possible to speculate on possible explanations for the missing decision-tree branches and the dichotomous options: (1) the goals of the decision are not clear or possibly confused with the decision-making problem; (2) there may still be no clear definition of what an option, a treatment option and a treatment are [108, 109]; (3) there may be an imbalance of power within different branches of medicine or between allopathic medicine and non-allopathic medicine. These could become the subject of further studies and will be briefly explained below.

First, options (treatment options, in this case) are defined with respect to certain (health) goals. Different goals lead to different treatment options [110]. Sometimes the goal is to cure an illness, other times the goal is to alleviate symptoms, other times the goal is to increase the quality of life and other times the goal is to make a value-congruent decision even if it leads to lower quality of life or death [63, 111, 112] or all of the above. Further research could investigate the extent to which the goals of the treatments, as opposed to the decision-problem are clearly stated in the PDAs.

Second, a potential treatment, as opposed to a validated treatment option, is generally accepted as a cure if it has been tested. However, it is not yet clear what kinds of potential treatments become the subject of testing. In the absence of a full list of potential treatments being tested, waiting to be tested and having been tested worldwide, it is possible that what constitutes a treatment depends on the way potential treatments are (accidentally or purposefully) selected to become the subject of testing and not just on the likelihood that they will cure the illness or increase quality of life, etc. It is possible that what makes a treatment is as much a representation of evidence as of its likelihood of belonging to the power or the mainstream group [70]. Thus, being a more likely representative of the power or mainstream group leads to a higher probability of being selected for testing. Irrespective of the way in which treatments get to become the subject of research testing or not (availability of funding, notoriety, etc.), some treatments will become a treatment option or not simply because there is research done about them or not, and not because it is really the best treatment available. While evidence-based medicine is beyond any doubt the desirable standard, reflecting on how treatments become valid options or not is also very important. One type of power imbalance is visible in the analysed PDA discourse where treatments belonging to allopathic medicine are individualized, while treatments belonging to CAM are not differentiated. For example, life-style changes are discussed as if all such changes are equal. Similarly, physical exercising is discussed as if all types of exercises are equal. Consequently, the gap between theory and practice may be further studied not only in terms of content, such as the one provided in this paper, but in terms of the power relations emerging from the content and of the stated goals. Further research may address these issues directly and propose solutions for them, particularly in the Shared Decision-Making conceptual framework to which PDAs belong.

Third, assuming there is no power imbalance and the goals are clear and differentiated from the decision problem, a decision-making option should represent a single treatment option with respect to the decision problem and the goal, not more. A treatment which solves side-effects of another treatment should be part of the option, but a treatment which is likely to have independent effects should not be clustered with others in ways which make it difficult to compare with the others. Further research may devise clear guidelines to help practitioners and PDA developers to differentiate between these practically relevant theoretical distinctions.

## Additional file

Additional file 1: Top-down and Bottom-up variables (XLSX 29 kb)

## Data Availability

The dataset supporting the conclusions of this article is included in the Additional file 1 of this article.

## Abbreviations

CAM: Complementary and Alternative Medicine
GRADE: Grading of Recommendations Assessment, Development and Evaluation
IPDAS: International Patient Decision Aids Standards
PDA: Patient Decision Aid
PDAs: Patient Decision Aids
PDF: Portable Document Format
PIR: Patient Information Resources
PS: Personal Stories
SDM: Shared Decision-Making
